# Long-Term Follow-Up of Targeted Biopsy Yield (LOFTY Study) in Ulcerative Colitis Surveillance Colonoscopy

**DOI:** 10.3390/jcm9072286

**Published:** 2020-07-18

**Authors:** Keisuke Hata, Soichiro Ishihara, Yoichi Ajioka, Keiichi Mitsuyama, Kenji Watanabe, Hiroyuki Hanai, Reiko Kunisaki, Hiroshi Nakase, Keiji Matsuda, Ryuichi Iwakiri, Nobuyuki Hida, Shinji Tanaka, Yoshiaki Takeuchi, Masaru Shinozaki, Noriyuki Ogata, Kentaro Moriichi, Fumihito Hirai, Kenichi Sugihara, Tadakazu Hisamatsu, Yasuo Suzuki, Mamoru Watanabe, Toshifumi Hibi

**Affiliations:** 1 Department of Surgical Oncology, The University Tokyo, 7-3-1 Hongo, Bunkyo-ku, Tokyo 113-8655, Japan; khata-tky@umin.ac.jp; 2 Division of Molecular and Diagnostic Pathology, Niigata University Graduate School of Medical and Dental Sciences, 1-757 Asahimachi-dori, Chuo-ku, Niigata 951-8510, Japan; ajioka@med.niigata-u.ac.jp; 3 Inflammatory Bowel Disease Center, Division of Gastroenterology, Department of Medicine, Kurume University School of Medicine, 67 Asahi-machi, Kurume, Fukuoka 830-0011, Japan; ibd@med.kurume-u.ac.jp; 4 Center for Inflammatory Bowel Disease, Division of Internal Medicine, Hyogo College of Medicine, 1-1, Mukogawa-cho, Nishinomiya, Hyogo 663-8501, Japan; ke-watanabe@hyo-med.ac.jp (K.W.); hidan@hyo-med.ac.jp (N.H.); 5 Center for Gastroenterology & IBD Research Hamamatsu South Hospital, 26 Shirowa-cho, Minami-ku, Hamamatsu 430-0846, Japan; flw-1013@topaz.plala.or.jp; 6 Inflammatory Bowel Disease Center, Yokohama City University Medical Center, 4-57 Urafune, Minami-ku, Yokohama 232-0024, Japan; kunisakireiko@gmail.com; 7 Department of Gastroenterology and Hepatology, Sapporo Medical University School of Medicine, S-1, W-16, Chuo-ku, Sapporo 060-8543 Japan; hiropynakase@gmail.com; 8 Department of Surgery, Teikyo University School of Medicine, 2-11-1 Kaga, Itabashi-Ku, Tokyo 117-0033, Japan; keiji@med.teikyo-u.ac.jp; 9 Faculty of Medicine, Saga University, 5-1-1, Nabeshima, Saga 849-8501, Japan; iwaryu9451.iwa@gmail.com; 10 Department of Endoscopy, Hiroshima University Hospital, 1-2-3 Kasumi, Minami-ku, Hiroshima 734-8551, Japan; colon@hiroshima-u.ac.jp; 11 Department of Medicine, Division of Gastroenterology, Showa University School of Medicine, 1-5-8, Hatanodai, Shinagawa-ku, Tokyo 142-8666, Japan; takeyoshi@east.cts.ne.jp; 12 Department of Surgery, the Institute of Medical Science, the University of Tokyo, Tokyo 108-8639, Japan; mshino@ims.u-tokyo.ac.jp; 13 Digestive Disease Center, Showa University Northern Yokohama Hospital, 35-1 Chigasaki-chuo, Tsuzuki Yokohama 224-8503, Japan; n.ogata@hotmail.co.jp; 14 Division of Gastroenterology and Hematology/Oncology, Department of Medicine, Asahikawa Medical University, Asahikawa 078-8510, Japan; morimori@asahikawa-med.ac.jp; 15 Department of Gastroenterology and Medicine, Fukuoka University Faculty of Medicine, 7-45-1 Nakanuma, Jonan-ku, Fukuoka 814-0180, Japan; fuhirai@cis.fukuoka-u.ac.jp; 16 Tokyo Medical and Dental University, 1-5-45 Yushima, Bunkyo-ku, Tokyo 113-8519, Japan; sugi.srg2@gmail.com; 17 Department of Gastroenterology and Hepatology, Kyorin University School of Medicine, 6-20-2 Shinkawa, Mitaka-shi, Tokyo 181-8611, Japan; thisamatsu@ks.kyorin-u.ac.jp; 18 Inflammatory Bowel Disease Center, Toho University Sakura Medical Center, 564-1 Shimoshizu, Sakura, Chiba 285-8741, Japan; yasuo-suzuki@sakura.med.toho-u.ac.jp; 19 Department of Gastroenterology and Hepatology, Advanced Research Institute, Tokyo Medical and Dental University, 1-5-45 Yushima, Bunkyo-ku, Tokyo 113-8519, Japan; mamoru.gast@tmd.ac.jp; 20 Center for Advanced IBD Research and Treatment, Kitasato Institute Hospital, Kitasato University, 5-9-1 Shirokanedai, Minato-ku, Tokyo 108-8642, Japan; thibi@insti.kitasato-u.ac.jp

**Keywords:** colonoscopy, colorectal neoplasms, inflammatory bowel disease, follow-up studies

## Abstract

We previously performed a randomized controlled trial (RCT) comparing targeted and random biopsy in neoplasia detection in patients with ulcerative colitis (UC), which showed the short-term effectiveness of targeted biopsy with one-time colonoscopy. In this retrospective cohort study, we investigated the long-term effectiveness of targeted biopsy in tertiary care hospitals, using the follow-up data from patients with UC for ≥ 8 years who had enrolled in the initial RCT. The primary outcome was death from colorectal cancer (CRC). Secondary outcomes were advanced neoplasia (CRC or high-grade dysplasia) and colectomy due to neoplasia after the RCT. We compared these outcomes between target and random groups. Data on 195 of the 221 patients (88.2%) enrolled in the previous RCT were collected from 28 institutions between 2008 and 2019. No patients died of CRC in either group, with a median 8.8-year follow-up demonstrating a robustness for targeted biopsy in terms of CRC death prevention. Advanced neoplasia was detected in four and three patients in the target and random groups, respectively. Colectomy was required due to neoplasia in three patients in each group. The chance of developing CRC in patients with a negative colonoscopy was low, and the targeted biopsy appeared effective in this population. Conversely, patients found with low-grade dysplasia at initial RCT have 10-fold higher risk of progression to high-grade dysplasia and/or CRC. Ten extracolonic malignancies were observed during the follow-up, resulting in four deaths. Panchromoendoscopy was used only in 4.6% and targeted biopsy was only performed in 59.1% of colonoscopies. We recommend targeted biopsy rather than > 33 random biopsies in real-world settings under adequate observation by specialists.

## 1. Introduction

Ulcerative colitis (UC) is an inflammatory bowel diseases (IBD), manifesting as chronic inflammation of the colorectum. Although medications such as 5-aminosalicylic acid, steroids, immunomodulators, and biological agents alleviate the inflammation, they cannot completely cure the disease [1]. Patients with long-standing UC are at increased risk for the development of colorectal cancer (CRC). Surveillance colonoscopy is recommended for those with extensive and left-sided UC, offering better survival than symptomatic colonoscopy [2,3,4,5]. In addition to CRC, patients with IBD are at a higher risk for extracolonic malignancies [6], which is yet another concern. Although the use of thiopurines in these patients may increase the risk of lymphoproliferative diseases and non-melanoma skin cancers in Caucasian populations [7,8], such an effect may not be extrapolated to the Asian population because of racial differences affecting the incidence of these malignancies [9].

We previously conducted a randomized controlled trial (RCT) comparing the effectiveness of targeted and random biopsies to detect neoplasia in patients with UC [10]. Although, in said RCT, the neoplasia detection rate was similar between the two biopsy methods, the trial was a cross-sectional study that assessed the results obtained from one-time colonoscopy. Thus, lesions may be missed by using targeted biopsy methods, which possibly results in advanced CRC. Although current guidelines recommend targeted biopsy rather than random biopsy [4,5], a recent retrospective study has suggested additional random biopsies in high-risk patients such as those with a personal history of neoplasia, concomitant primary sclerosing cholangitis (PSC), or a tubular colon during colonoscopy [11]. This is partly because most prospective studies have only investigated short-term results, and long-term follow-up studies are lacking. Therefore, in this study, we conducted a retrospective cohort study using endoscopic data in patients enrolled in our initial RCT to determine the incidence of CRC and the long-term effectiveness of targeted biopsy. Real-world surveillance methods and the incidence of extracolonic malignancies were also investigated in this cohort.

## 2. Methods

### 2.1. Initial Randomized Controlled Trial

The initial RCT compared targeted and random biopsy in patients with UC [10]. The trial is registered at the UMIN Clinical Trial Registry as UMIN000001608 (http://www.umin.ac.jp/ctr/index-j.htm). Briefly, the original RCT was conducted using 1:1 randomization to the target and random groups. Patients with UC for at least 8 years were enrolled. Data from 114 patients in the target group and 107 patients in the random group from 34 institutions were finally analyzed. Four random biopsies were taken per 10-cm length in addition to targeted biopsy in the random group, and one rectal random biopsy was taken in addition to targeted biopsy in the target group. Biopsy methods after the RCT were based on the endoscopist’s discretion. The primary outcome of the preceding RCT was the number of biopsy samples with neoplasia per one-time colonoscopy.

### 2.2. Data Collection

This was an observational, retrospective, multicenter follow-up study after the initial RCT. Patient data were deidentified and names were converted to case identification numbers in each institution at the time of the initial RCT. In the present study, data from October 2008 to September 2019 were retrospectively collected from medical charts in each institution and sent to the University of Tokyo after re-anonymization using the previously defined case identification numbers. Collected data were then merged with the data of the initial RCT according to the case identification numbers. Collected data included information on survival, colectomy, endoscopic resection, subsequent data, on colonoscopy (the number of biopsies, pathology, the use of chromoendoscopy), medication [5-aminosalicylic acid, anti-tumor necrosis factor (TNF), immunomodulator, steroid, apheresis] at the time of RCT, smoking history, and history of malignancy. In terms of pathology, we utilized the initial pathological data from the RCT for the colonoscopies performed at the RCT, and those from each institution for follow-up colonoscopies. For those with CRC, data on cancer stage were also collected.

The primary outcome of this study was CRC-specific mortality between the target and random groups. The secondary outcomes included overall survival, overall colectomy rate, colectomy rate for neoplasia, and the incidence of extracolonic malignancy during the follow-up. We compared the incidence of invasive CRC and the colectomy rate due to neoplasia between patients who were diagnosed as having neoplasia at the initial RCT (neoplasia group) and those who were not diagnosed as having neoplasia (non-neoplasia group) as well as between the target and the random groups. We also investigated the cumulative rate of advanced neoplasia [CRC or high-grade dysplasia (HGD)] in patients who had no dysplasia and those who were found to have low-grade dysplasia (LGD) at the time of the initial RCT.

Finally, to clarify the real-world method of surveillance, we collected data on the use of chromoendoscopy [panchromoendoscopy, partial, only for suspicious area, or white light endoscopy (WLE) only], random biopsy, the number of biopsies, and colonoscopy (CS) intervals.

### 2.3. Statistical Analysis

All statistical analyses were performed using EZR version 1.38 (Saitama Medical Center, Jichi Medical University, Saitama, Japan), which is a graphical user interface for R (The R Foundation for Statistical Computing, Vienna, Austria) [12]. Pearson’s chi-square or Fisher exact test was used for categorical variables, and Student’s t-test or Kruskal–Wallis test was used for continuous variables. The time-to-event analysis was performed and the cumulative risk was estimated using the Kaplan–Meier method. Hazard ratios were calculated using the Cox regression test. The start of the follow-up period was set as the date of colonoscopy at the initial RCT. For the time-to-event analyses of colectomy for neoplasia, invasive CRC, and advanced neoplasia analyses, patients were censored at colectomy or death. For colectomy analysis, patients were censored at death. Patients who did not undergo either colectomy or colonoscopy after the RCT were excluded from the time-to-event analyses for invasive CRC and advanced neoplasia. For the patient-year method, binomial confidence interval was used to estimate 95% confidence interval (CI) for the proportion.

### 2.4. Ethics

This study was approved by the ethics committee of the University of Tokyo (2018143NI-(1)). Informed consent was obtained from all the patients at the time of the initial RCT [10] and was waived in the present study due to the retrospective nature of the study.

## 3. Results

Survival data could retrospectively be obtained from 195 (88.2%) of the 221 patients included in the final analysis set of the initial RCT. Background characteristics of the patients are listed in Table 1. The median follow-up period was 105 months (interquartile range 89–111 months) accounting for 1521 patient-years for survival data. The median number of colonoscopies after the RCT was 6 (interquartile range: 3.75–8), and the median interval between colonoscopy sessions was 12.4 months (interquartile range: 11.9–19.4).

### 3.1. Mortality and Cause of Death

Six patients died, yielding a five-year overall survival rate of 96.7% (95% CI: 92.7–98.5%) after the previous RCT. This corresponded to 3.9 deaths per 1000 patient-years (6/1521). Notably, none of the deaths was attributed to CRC, indicating the effectiveness of surveillance colonoscopy in both target and random groups in this cohort. Four died of extracolonic cancer (cancer of unknown primary, lung cancer, cholangiocarcinoma, and parotid cancer; Table 2), and two died of non-cancer causes (suicide and pneumonia).

### 3.2. Colorectal Cancer Development and Fate of Dysplasia Detected in the RCT

**Figure 1:**
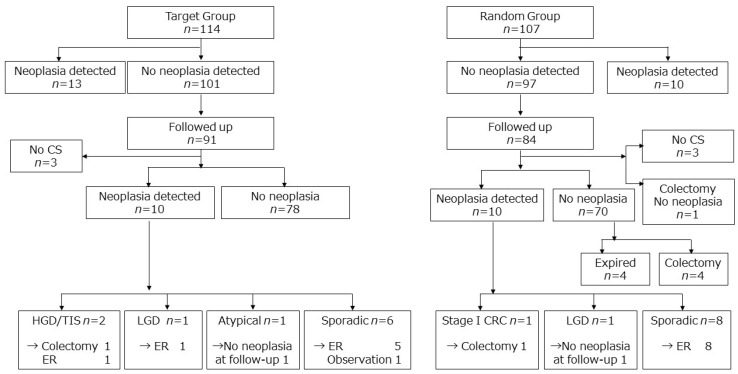
Follow-up results of patients without neoplasia during the randomized controlled study. Only one patient from the random group developed invasive colorectal cancer. Abbreviation: CRC, colorectal cancer; CS, colonoscopy; ER, endoscopic removal; HGD, high-grade dysplasia; LGD, low-grade dysplasia; TIS, tumor in situ.

**Figure 2:**
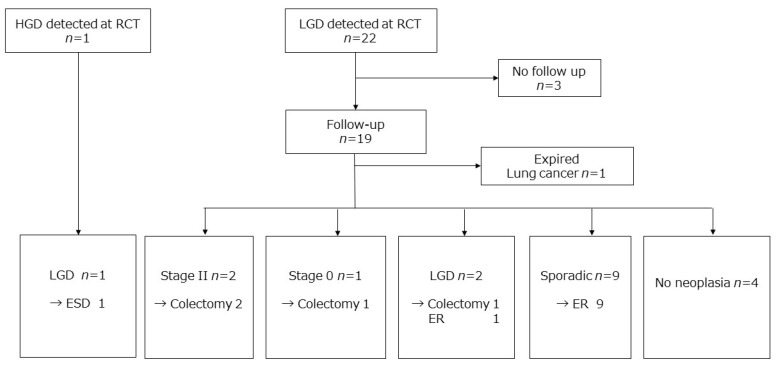
Outcome of patients diagnosed with neoplasia during the randomized controlled study. HGD—high-grade dysplasia; LGD—low-grade dysplasia; ESD—endoscopic submucosal dissection; ER—endoscopic removal.

**Figure 3:**
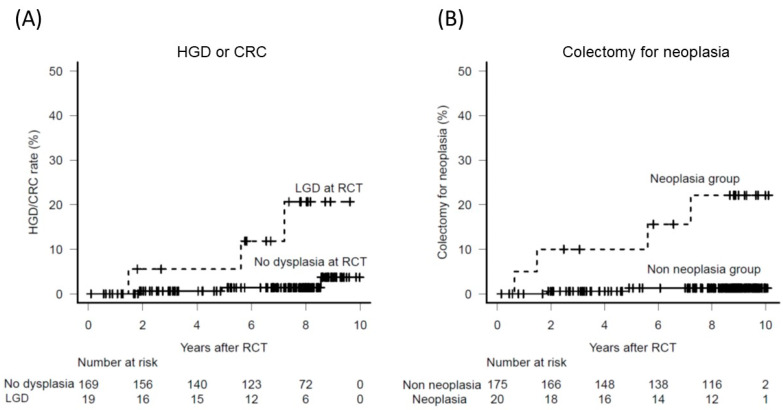
(**A**) The cumulative rate of advanced neoplasia was significantly higher than in the non-neoplasia group (hazard ratio 10.0, 95% CI: 2.0–49.7; *p* = 0.005). (**B**) The cumulative rate of colectomy for neoplasia was significantly higher among patients who were diagnosed as having neoplasia at the RCT than those without neoplasia at the RCT (hazard ratio 9.3, 95% CI: 1.9–46.0; *p* = 0.007). Abbreviations: HGD, high-grade dysplasia; LGD, low-grade dysplasia.

The major concern of surveillance colonoscopy is the possibility of missed cases of advanced interval CRC. Advanced neoplasia was detected in four and three patients in the target and random groups, respectively (Table 3). The follow-up results among the patients in the non-neoplasia group are shown in Figure 1. Invasive CRC (stage I) was found only in one patient (0.77 per 1000 patient-years) and advanced neoplasia (HGD or CRC) in three patients (2.3 per 1000 patient-years). Invasive CRC was found in one patient in the random group, while advanced neoplasia was detected in two patients in the target group. Figure 2 presents the follow-up results among the patients in the neoplasia group. Among the 19 patients with LGD at the initial RCT, the cumulative rate of advanced neoplasia was significantly higher than in the non-neoplasia group with a hazard ratio 10.0 (95% CI: 2.0–49.7; *p* = 0.005) (Figure 3A).

In the target group, among the 101 patients without neoplasia during the initial RCT, data on 91 patients could be obtained. Ten patients (11.0%) developed neoplasia during the follow-up. One patient underwent colectomy, and the final pathological diagnosis was HGD. Another patient was found to have cancer in colonoscopic biopsies on two occasions; however, no neoplastic change was detected in endoscopic submucosal dissection specimens. The other six patients were treated by endoscopic removal of the tumor (Figure 1).

In the random group, data on 84 patients without neoplasia during the RCT could be obtained. Four did not receive follow-up colonoscopy, out of which one underwent colectomy due to inflammation. Ten (12.5%) of the remaining 80 patients developed neoplasia during the follow-up, and one underwent colectomy with the final pathological diagnosis being stage I CRC. The other eight patients were managed by endoscopic removal of the tumor. Four more patients underwent colectomy due to inflammation. Four patients died (Figure 1).

**Figure 4:**
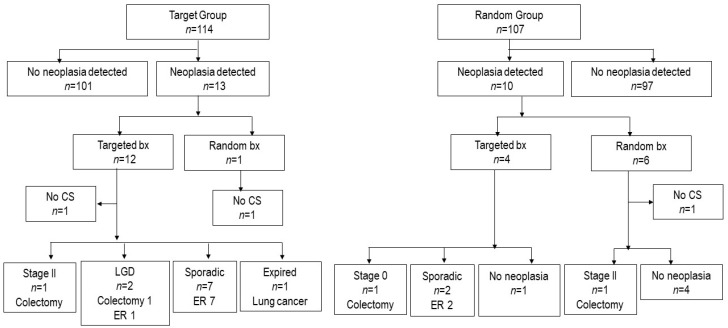
The fate of neoplasia detected at RCT. Abbreviation: bx- biopsy; CS- colonoscopy; ER- endoscopic removal; LGD- low-grade dysplasia.

In the initial RCT, 22 patients were diagnosed with LGD and one with HGD. Of the total 23 patients, 20 patients were followed up in the present study. During the follow-up, four patients (20%) underwent colectomy due to neoplasia: two, CRC; one, intramucosal carcinoma; and one, LGD. Detailed information on the outcomes for the patients in the target and random groups is shown in Figure 2, Figure 3B and Figure 4; and Table 4.

### 3.3. Colectomy Rate

Colectomy was required to treat neoplasia in three patients in each of the target and random groups. Additionally, five patients in the random group required colectomy due to inflammation with no evidence of dysplasia found on the histopathological assessment of the surgical specimens. The cumulative overall colectomy rate after the initial RCT was 4.3% (95% CI: 2.2–8.5%) after 5 years and 6.3% (95% CI: 3.5–11.1%) after 8 years, corresponding to 7.5 per 1000 patient-years. The cancer stage was II in two patients, I in one patient, HGD/intramucosal carcinoma in two patients, and LGD in one patient. Four of the 20 patients diagnosed with neoplasia during the initial RCT underwent colectomy due to neoplasia, and only two of the 189 patients without any evidence of neoplasia during the initial RCT underwent colectomy due to neoplasia (Figure 3B). The colectomy rate due to neoplasia was significantly higher in patients diagnosed with neoplasia during the initial RCT with a hazard ratio of 9.3 (95% CI: 1.9–46.0; *p* = 0.007).

### 3.4. Extra-Colonic Cancer

Extracolonic cancer was relatively frequently observed. A total of ten patients developed extracolonic malignancy after the RCT, as listed in Table 2. The observation time was median 105 months and 1,521 patient-years. Six patients had a history of extracolonic malignancies before the RCT (testis, prostate, cervix, Barrett esophagus, pharynx and lung, and breast). An immunomodulator was prescribed within 1 year prior to the RCT in 55 patients, of which only one developed breast cancer. No cases of lymphoma, leukemia, or skin cancer was observed. Anti-TNFα antibody was administered only in five patients before randomization, as the use of anti-TNF antibody for UC was approved in Japan in 2010, and patient enrollment for the initial RCT was between 2009 and 2011.

### 3.5. Real-World Surveillance Method After the RCT

After the initial RCT, biopsy methods and colonoscopy intervals were at the endoscopists’ discretion, but most endoscopists turned out to have followed the Japanese clinical practice guidelines [3], with the median number of biopsy samples and colonoscopy interval being three and 12.4 months, respectively. Data on colonoscopic follow-up were collected from 186 patients. A total of 4106 biopsies were performed in 1085 colonoscopies, accounting for a median of three biopsy samples (interquartile range: 1–6) per colonoscopy, including the assessment for microscopic inflammation. The number of biopsy samples per CS is shown in Table 4. More than 33 biopsy samples were obtained from one patient who had been diagnosed as having dysplasia on random biopsies taken in the initial RCT. At least one random biopsy sample was performed in 41% of colonoscopies; however, ten or more biopsy samples were taken only in 7.6% of colonoscopies. The information on chromoendoscopy was also obtained from 1077 colonoscopy sessions as listed in Table 4. Panchromoendoscopy was used only in 50 colonoscopies (4.6%), and dye spraying was partially applied in 295 colonoscopies (27.4%). Dye spraying was used only for suspicious lesions detected using WLE in 169 colonoscopies (15.7%), and was never used in 563 colonoscopies (52.3%). In the subsequent colonoscopy among the 18 patients diagnosed with dysplasia in the initial RCT, panchromoendoscopy was used in 2 (11%), and dye spraying was applied partially in 9 (50%) and only for suspicious lesions in 2 (11%).

At the time of RCT, approximately three-fourths of the institutions involved in the study utilized high-definition colonoscopy in all instances, while the rest of the institutions used it whenever possible. Nowadays, most institutions use high-definition endoscopes all the time.

## 4. Discussion

To the best of our knowledge, this is the first study to demonstrate the long-term effectiveness of targeted biopsy in surveillance colonoscopy of patients with UC. In the initial RCT comparing targeted and random biopsy [10], overlooked cases, particularly in the target group, were a concern because retrospective studies had reported a positive role for random biopsies [2,11]. However, no patients died from CRC in either the target or random group, indicating the robustness of targeted biopsy in this cohort. One patient who died from cancer of unknown primary was originally in the random group, and no dysplasia was detected at the time of the initial RCT. In addition, the colectomy rates due to neoplasia and the incidence of advanced neoplasia were comparable between target and random groups, although the number of events was small.

The rate of colectomy due to neoplasia was higher among the patients in the neoplasia group than that among the patients in the non-neoplasia group. By contrast, many patients received endoscopic resection with a clinical diagnosis of sporadic adenoma or confined dysplasia. In the present study, the HGD/CRC incidence was 0.26 per 100 patient-years (95% CI: 0.054–0.76) among patients with a negative colonoscopy, and it was 2.5 per 100 patient-years (95% CI: 0.52–7.2) among those with LGD during the initial RCT. A recent study has reported that one positive colonoscopy predicted HGD or CRC incidence of 0.29 to 0.76/100 patient-years, and consecutive negative colonoscopy had no positive result at surveillance biopsy [13]. Another study from St. Marks revealed that 19% of the patients with LGD developed CRC after a follow-up of 48 months [14]. We observed two invasive CRCs and one intramucosal cancer in the 20 cases with LGD, which may be slightly lower than that reported in the St. Marks’ cohort; however, there was a significantly higher advanced neoplasia rate in patients with LGD than those without any signs of neoplasia during the RCT. Although the standard treatment of choice after the detection of HGD and multi-focal or consistent LGD is restorative proctocolectomy and ileal pouch-anal anastomosis [2,15,16], the SCENIC statements recommend careful surveillance colonoscopy after complete removal of the dysplastic lesions rather than colectomy [17]. The follow-up colonoscopy is planned in 3 to 6 month intervals, except for endoscopic removal of the suspected sporadic adenoma, as the risk of interval CRC is not considered high in these cases [18].

Extraintestinal malignancy is also an important issue in the management of patients with IBD. While a previous meta-analysis showed no overall increase in extraintestinal malignancy in IBD [19], a recent study reported a higher incidence for extraintestinal malignancy among patients with IBD than in the general population [6]. In the present study, six patients died during the follow-up, four of which were due to extracolonic malignancies. A total of ten patients (7%) developed extracolonic malignancies after the initial RCT. We did not observe any difference in the origin of the primary tumor and the age at diagnosis in our cohort and the general population. The primary sites where more than two patients developed cancer were the lung, breast, and kidney, which was consistent with the results of a previous study [20]. No patient developed lymphoma, leukemia, or skin cancer during the follow-up period, while in Western countries, the risk of lymphoma is reportedly high, especially in patients on thiopurine [21]. Racial differences may exist in terms of lymphoma susceptibility; our findings may be consistent with those of previous studies [9,22]. A retrospective survey showed only 12 (0.11%) of the 10,500 Japanese patients with UC experienced hematologic malignancies [9]. Another recent large-scale study, using a nationwide administrative database in Japan, also reported only 103 (0.14%) cases of Non-Hodgkin lymphoma in 75,673 patients with UC with no increase in the incidence of lymphoma among patients on thiopurines [22].

Targeted biopsy using chromoendoscopy is a recommended method in Western countries. However, most endoscopists in the present study adopted targeted biopsy with WLE and applied chromoendoscopy only for suspected lesions. Such preference in real-life practice among Japanese specialists has already been reported [23,24], with dysplasia detection rates being almost identical to that reported in studies using chromoendoscopy in Western countries [10]. Although a couple of controlled studies demonstrated the benefit of panchromoendoscopy a decade ago [25,26], a recent multicenter retrospective study from the Netherlands has demonstrated that chromoendoscopy was less used and did not increase the dysplasia detection rate compared with WLE [27]. In addition, a recent Korean RCT comparing high-definition chromoendoscopy and high-definition WLE did not reveal any difference in dysplasia detection rate between the two methods [28]. Although the SCENIC statements suggests the use of chromoendoscopy rather than WLE in the high-definition setting with conditional recommendation [17], such recommendation was based on a small observational study. Although a recent network meta-analysis implied that full spectrum high-definition WLE and FICE may be the first-line approach, high-definition WLE alone was not inferior to any of these modalities [29]. Therefore, high-definition WLE might be considered a reliable method in detecting dysplasia, and could be an alternative to Western standards [30].

This study has several limitations. First, the pathological evaluation of specimens in the initial RCT was centralized, with three expert gastrointestinal pathologists confirming the results, while the present retrospective study relied on the pathological reports from each institute. Diagnosing LGD is difficult in many ways. Inter- and intra-observer variances exist due to the changes caused by inflammation [31]. In addition, sporadic adenoma and colitis-associated “dysplasia” are sometimes difficult to distinguish, and integrated interpretations are often needed for clinical decision-making [32]. Therefore, we set the endpoints of colectomy due to neoplasia, advanced neoplasia (HGD or CRC), and invasive CRC to avoid any differences in the diagnostic criteria among the pathologists from different institutes. Most neoplasias detected in each institute after the initial RCT were reportedly sporadic adenomas and were managed by endoscopic removal. Besides, although pathological inflammation has been reported to be a risk factor for CRC in patients with UC [33], we did not assess pathological inflammation and its association with CRC development. Second, a study recommended random biopsy for patients with PSC [11], whereas no concurrent PSC cases were found in the present study. The initial RCT did not intend to exclude PSC, and this was probably due to the low incidence of PSC and low comorbidity of IBD in Japan [34]. Thus, we could not conclude the effectiveness of targeted biopsy for patients with PSC in the present study. Third, biases were not completely excluded because the data were retrospectively collected after the RCT. In addition, the initial RCT only included patients with inactive disease, as defined by the inclusion criteria, which may lead to lower colectomy rate than that in other cohort studies assessing CRC rate [33]. Considering that a risk factor for CRC development in patients with UC includes macroscopic and microscopic inflammation [35,36], we might have underestimated the risk of CRC by just looking at quiescent patients.

In conclusion, no patients died of CRC after the initial RCT comparing targeted and random biopsy in patients with UC, with median 8.8-year follow-up, demonstrating a robustness of targeted biopsy in preventing death due to CRC. The targeted biopsy strategy appeared effective for patients with a negative colonoscopy reported at the initial RCT, because the possibility of developing CRC in these patients was small, provided they were compliant with surveillance colonoscopy in tertiary care hospital settings. Conversely, those with LGD at the initial RCT had a significantly higher progression rate to HGD and/or CRC. Based on this LOFTY study, we recommend targeted biopsy rather than random biopsy with > 33 samples in real-world settings under adequate observation by specialists.

## Figures and Tables

**Table 1:** Background characteristics of followed-up patients in both groups.

Characteristics	Random Group	Target Group	*p*-Value
**Followed-up cases**	*n* = 93	*n* = 102	
Neoplasia at RCT	11	9	
No neoplasia at RCT	82	93	
**Age, y, mean (SD)**	48.3 (13.4)	49.8 (14.0)	0.447
Sex			
Female	29 (31.2%)	39 (38.2%)	0.367
Male	64 (68.8%)	63 (61.8%)	
**Extent of UC**			
Total colitis	63 (67.7%)	57 (55.9%)	0.207
Left-sided colitis	25 (26.9%)	39 (38.2%)	
Others	5 (5.4%)	6 (5.9%)	
**Primary sclerosing cholangitis**	0 (0%)	0 (0%)	
**UC duration at RCT**	16.4 ± 6.9	15.8 ± 6.5	0.523
**Smoking history**			
Never smoked	52 (55.9%)	62 (60.8%)	0.19
Current smoker	8 (8.6%)	4 (3.9%)	
Ex-smoker	9 (9.7%)	17 (16.7%)	
Unknown	24 (25.8%)	19 (18.6%)	
**Medication at RCT**			
5 ASA	86 (92.5%)	101 (99.0%)	0.029
Steroid	13 (14.1%)	15 (14.7%)	1
Apheresis	12 (12.9%	20 (19.6%)	0.247
Immunomodulator	29 (31.2%)	26 (25.5%)	0.427
Anti-TNFα	5 (5.4%)	2 (2.0%)	0.261
**Number of biopsies, median (IQR)**			
At RCT	36 (29,37)	3 (2, 8)	<0.001
After RCT	3 (1, 6)	3 (1, 6)	0.204
**Follow-up, y, mean (range)**	8.7 (0.14–10.0)	8.8 (0.98–10.1)	0.902

ASA, 5-aminosalicylic acid; CRC, colorectal cancer; IQR, interquartile range; RCT, randomized controlled trial; SD, standard deviation; TNF, tumor necrosis factor.

**Table 2:** Extracolonic malignancies observed after the randomized controlled study.

Age	Sex	Smoking	Anti-TNF *	Thiopurine *	Primary Site	Status
60s	M	Current	−	−	Unknown primary	Dead
50s	M	Current	−	−	Bile duct	Dead
60s	M	Ex	−	−	Parotid gland	Dead
70s	M	Ex	−	−	Lung	Dead
80s	M	Ex	−	−	Lung	Alive
70s	M	Never	−	−	Pancreas	Alive
50s	F	Never	−	+	Breast	Alive
60s	M	Never	−	−	Breast	Alive
70s	M	Never	−	−	Kidney	Alive
80s	F	Unknown	−	−	Kidney	Alive

TNF, tumor necrosis factor, * The use of anti-TNF or thiopurines was recorded for 1 year prior to RCT enrollment.

**Table 3:** Cases with high-grade dysplasia or cancer at some point.

Group	Sex	Age at RCT	Pathology at RCT	Final Pathology	Interval after RCT (years)	Procedure	Location ^$^ and Morphology at CS	Remarks
Random	Female	50 s	LGD	CRC (T4N0M0)	5.6	Colectomy after ESD	R, 0-IIa	Same location †
Random	Female	40 s	LGD	Intramucosal Ca	1.5	Colectomy after EMR	R, 0-IIa+Is	Additional surgery ‡
Random	Male	60 s	neg	CRC (T1bN0M0)	4.9	Colectomy after EMR	T, 0-Isp	Additional surgery ‡
Target	Female	40 s	HGD	LGD	0.6	ESD	D, 0-IIa, 0-IIa	Two synchronous lesions §
Target	Male	40 s	LGD	CRC (T3N0M0)	7.2	Colectomy	S, 0-IIa	Progression? §§
Target	Male	50 s	neg	HGD	1.8	Colectomy	R, 0-IIb	
Target	Female	30 s	neg	Intramucosal Ca *	8.6	ESD	R, 0-IIb	

CRC, colorectal cancer; CS, colonoscopy; EMR, endoscopic mucosal resection; ESD, endoscopic submucosal dissection; HGD, high-grade dysplasia; LGD, low-grade dysplasia; neg, negative for dysplasia; RCT, randomized controlled trial; ^$^ R, rectum; S, sigmoid colon; D, descending colon; T, transverse colon; * Intramucosal carcinoma was reported twice with biopsy, but diagnostic ESD specimens were negative for neoplasia. Thus, careful surveillance was continued. † Recurrence or metachronous cancer in the same location after “complete” pathological endoscopic resection, as the patient initially refused colectomy. ‡ EMR was performed for the diagnostic purpose, and no evidence of residual neoplasia was found on surgical specimens after additional surgical resection. § Diagnostic ESD revealed the lesion was LGD, thus careful surveillance was chosen with frequent intervals. §§ LGD lesion was detected at the time of the initial RCT, but not in the five subsequent colonoscopies. HGD was detected seven years after the initial RCT.

**Table 4:** Real-life method of surveillance.

Variable	*n*	%
**Number of colonoscopies**	1085	
**Biopsy method**		
Targeted biopsy only	581	59.1%
Targeted plus random biopsy	402	40.9%
**Number of biopsy specimens**		
<10	1002	92.3%
10–19	80	7.4%
20–33	2	0.2%
>34	1	0.1%
**Chromoendoscopy**		
Panchromoendoscopy	50	4.6%
Specific area	295	27.4%
Targeted area only	169	15.7%
No dye spray	563	52.3%

## Abbreviations

CI	confidence interval
CRC	colorectal cancer
CS	colonoscopy
HGD	high-grade dysplasia
LGD	low-grade dysplasia
LOFTY	LOng-term Follow-up of Targeted biopsy Yield
PSC	primary sclerosing cholangitis
RCT	randomized controlled trial
TNF	tumor necrosis factor
WLE	white light endoscopy
